# TRIM32-TAX1BP1-dependent selective autophagic degradation of TRIF negatively regulates TLR3/4-mediated innate immune responses

**DOI:** 10.1371/journal.ppat.1006600

**Published:** 2017-09-12

**Authors:** Qing Yang, Tian-Tian Liu, Heng Lin, Man Zhang, Jin Wei, Wei-Wei Luo, Yun-Hong Hu, Bo Zhong, Ming-Ming Hu, Hong-Bing Shu

**Affiliations:** 1 Medical Research Institute, School of Medicine, Wuhan University, Wuhan, China; 2 Department of Cell Biology, College of Life Sciences, Wuhan University, Wuhan, China; University of Toronto, CANADA

## Abstract

Toll-like receptor (TLR)-mediated signaling are critical for host defense against pathogen invasion. However, excessive responses would cause harmful damages to the host. Here we show that deficiency of the E3 ubiquitin ligase TRIM32 increases poly(I:C)- and LPS-induced transcription of downstream genes such as type I interferons (IFNs) and proinflammatory cytokines in both primary mouse immune cells and in mice. *Trim32*^-/-^ mice produced higher levels of serum inflammatory cytokines and were more sensitive to loss of body weight and inflammatory death upon *Salmonella typhimurium* infection. TRIM32 interacts with and mediates the degradation of TRIF, a critical adaptor protein for TLR3/4, in an E3 activity-independent manner. TRIM32-mediated as well as poly(I:C)- and LPS-induced degradation of TRIF is inhibited by deficiency of TAX1BP1, a receptor for selective autophagy. Furthermore, TRIM32 links TRIF and TAX1BP1 through distinct domains. These findings suggest that TRIM32 negatively regulates TLR3/4-mediated immune responses by targeting TRIF to TAX1BP1-mediated selective autophagic degradation.

## Author summary

TLR3/4-mediated signaling needs to be effectively terminated to avoid excessive immune responses and harmful damages to the host. In this study, we provide genetic evidence to show that the E3 ubiquitin ligase TRIM32 negatively regulates TLR3/4-mediated innate immune and inflammatory responses. *Trim32*^-/-^ mice are more sensitive to the inflammatory death upon *Salmonella typhimurium* infection. We found that TRIM32-TAX1BP1-dependent selective autophagic degradation of the adaptor protein TRIF effectively turned off TLR3/4-mediated innate immune and inflammatory responses. Our findings reveal a novel mechanism for terminating innate immune and inflammatory responses mediated by TLR3/4.

## Introduction

The innate immune system is the first line of host defense against pathogen invasion. After detection of structurally conserved components of the invading pathogens by so-called pathogen recognition receptors (PRRs), the host cells initiate a series of signaling cascades which ultimately induce the transcription of downstream antiviral genes, such as type I interferons (IFNs) and inflammatory cytokines, to induce innate immune and inflammatory responses as well as facilitate adaptive immunity [1,2,3,4]. However, excessive immune and inflammatory responses cause tissue damages and serious diseases such as septic shock [5].

Toll-like receptors (TLRs) are evolutionarily conserved PRRs that play critical roles in host defense against various pathogens. TLRs contain an extracellular domain, a transmembrane domain, and a conserved cytoplasmic toll/IL-1 receptor (TIR) domain. Upon ligand stimulation, the TIR domains of TLRs mediate their homo- or hetero-dimerization [6], and act as platforms to recruit downstream TIR domain-containing adaptor proteins and other signaling molecules, leading to the activation of transcription factors such as IRF3 and NF-κB. These transcription factors collaborate to induce the transcription of a series of downstream antiviral genes [7]. Most TLRs except TLR3 and TLR4 signal through the TIR-containing adaptor MyD88. TLR3, which recognizes viral dsRNA and plays important roles in innate antiviral responses, signals through the TIR-containing adaptor TRIF but not MyD88 [8]. TLR4, which recognizes LPS of bacteria and is essential for innate and inflammatory responses to infected bacteria, signals through MyD88 to activate NF-κB and TRIF to activate NF-κB and IRF3 [9]. Double knockout of TRIF and MyD88 results in completely abolishment of LPS-induced activation of NF-κB, whereas TRIF-deficiency results in abolishment of LPS-induced activation of IRF3 [9].

Protein degradation is one of the main strategies which have been employed by host cells to inactivate proteins in biological processes. Autophagy is an essential homeostatic process by which damaged organelles, protein aggregates, and invading cytoplasmic microbes are sequestered in double-membraned autophagosomes and delivered to the lysosome for degradation [10]. There are growing evidences that autophagy can be highly selective [11]. Selective autophagy depends on the cargo receptors, including p62, TAX1BP1, NDP52 and so on, which are able to bind to special cargoes and dock onto the forming phagophores [11]. Certain selective autophagy receptors have been reported to be involved in regulation of immune responses. For examples, the cytosolic DNA viral sensor cGAS and intracellular *Salmonella typhimurium* can be degraded via p62- and TAX1BP1-dependent selective autophagy respectively [12,13]. Whether selective autophagy is involved in the regulation of other immune processes are unknown.

The tripartite motif-containing proteins (TRIMs) of E3 ubiquitin ligase families have been demonstrated to play critical regulatory roles in regulation of immune responses [14,15]. TRIM32 has been reported to mediate K63-linked polyubiquitination of MITA/STING and regulates innate immune responses to RNA and DNA viruses in human cell lines [7]. In this study, we generated TRIM32-deficient cells and *Trim32* knockout mice, and found that TRIM32 negatively regulated TLR3/4-mediated innate immune and inflammatory responses. Biochemical and cellular analysis revealed that TRIM32 mediated selective autophagic degradation of TRIF through TAX1BP1. Our findings suggest that TRIM32-TAX1BP1-dependent selective autophagic degradation of TRIF is an important negative regulatory mechanism of TLR3/4-mediated innate immune and inflammatory responses.

## Results

### TRIM32 negatively regulates TLR3/4-mediated signaling in primary mouse cells

**Fig 1:**
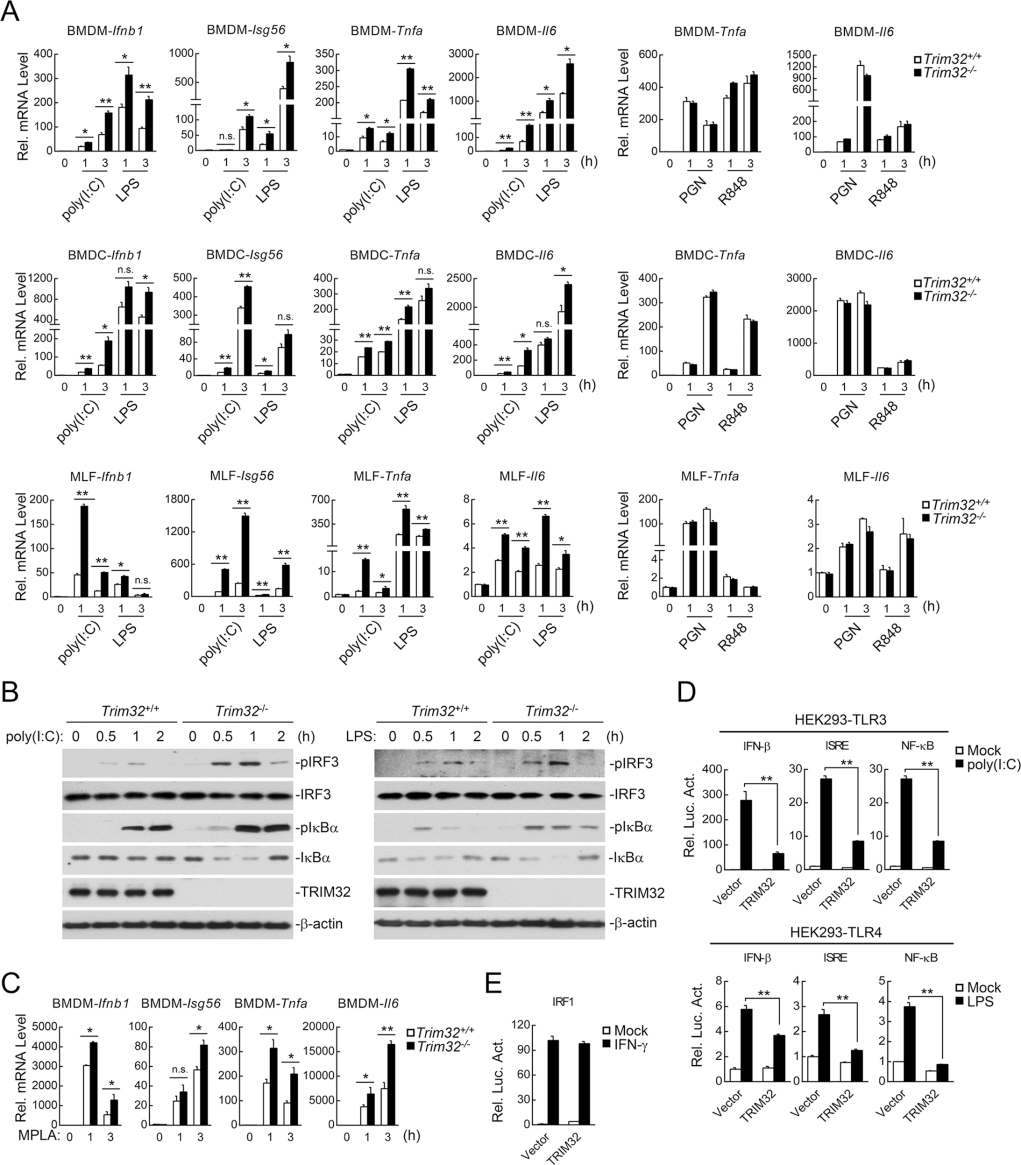
TRIM32-deficiency potentiates TLR3/4-mediated signaling. (A) Effects of TRIM32-deficiency on poly(I:C)-, LPS-, PGN-, or R848-induced transcription of *Ifnb1*, *Isg56*, *Tnfa* and *Il6* in BMDMs, BMDC and MLFs. *Trim32*^+/+^ and *Trim32*^-/-^ cells were stimulated with poly(I:C) (50 μg/ml), LPS (50 ng/ml), R848 (20 nM) or PGN (20 μg/ml) for the indicated times before qPCR was performed. (B) Effects of TRIM32-deficiency on poly(I:C)- and LPS-induced phosphorylation of IRF3 and IκBα in MLFs. Wild-type and *Trim32*^-/-^ MLFs were stimulated with poly(I:C) (100 μg/ml) or LPS (100 ng/ml) for the indicated times before immunoblots were performed with the indicated antibodies. (C) Effects of TRIM32-deficiency on MPLA-induced transcriptions of *Ifnb1*, *Isg56*, *Tnfa* and *Il6* genes in BMDMs. *Trim32*^*+/+*^ and *Trim32*^*-/-*^ BMDMs were stimulated with MPLA (100 ng/ml) for the indicated times before qPCR was performed. (D) Effects of TRIM32 on poly(I:C)- or LPS-triggered activation of IFN-β, ISRE and NF-κB. HEK293-TLR3 cells or HEK293-TLR4 cells (1x10^5^) were transfected with the indicated luciferase plasmid (0.1 μg) and an HA-tagged expression plasmid for murine TRIM32 (0.05 μg). Twenty hours after transfection, cells were treated with poly(I:C) (50 ng/mL) or LPS (100 ng/ml) for 10 hours before luciferase assays were performed. (E) Effects of TRIM32 on IFNγ-induced activation of the IRF1 promoter. HEK293 cells were transfected with the IRF1 promoter reporter plasmid (0.1 μg) and the indicated amounts of HA-tagged murine TRIM32 expression plasmids (0.05 μg). Twenty hours after transfection, cells were treated with IFNγ (100 ng/mL) or left untreated for 10 hours before luciferase assays were performed. n.s., Not Significant. *, p<0.05; **, p<0.01.

Previously, it has been demonstrated that TRIM32 mediates K63-linked polyubiquitination of MITA/STING and regulates virus-triggered induction of downstream antiviral genes in human cell lines [7]. To investigate the functions of TRIM32, we utilized TRIM32 gene knockout mice (S1A & S1B Fig). We found that TRIM32-deficiency had no marked effects on the mRNA levels of downstream antiviral genes *Ifnb1* and *Isg56* as well as inflammatory cytokine genes *Tnfa* and *Il6* induced by Sendi virus (SeV) or herpes simplex virus 1 (HSV-1) in mouse embryonic fibroblasts (MEFs), bone marrow-derived macrophages (BMDMs) and dendritic cells (BMDCs) (S1C Fig), suggesting that TRIM32 does not regulate virus-triggered signaling in primary mouse cells. However, we found that TRIM32-deficiency potentiated poly(I:C) (a synthetic dsRNA ligand for TLR3)- and LPS (a ligand for TLR4)- but not PGN (a ligand for TLR2)- or R848 (a ligand for TLR7)-induced transcription of downstream genes *Ifnb1*, *Isg56*, *Tnfa* and *Il6* in BMDMs, BMDCs and mouse lung fibroblasts (MLFs) (Fig 1A). Consistently, poly(I:C)- and LPS-induced phosphorylation of IRF3 and IκBα (hallmarks for IRF3 and NF-κB activation respectively) was dramatically increased in *Trim32*^-/-^ MLFs in comparison to their wild-type counterparts (Fig 1B). LPS has been reported to induce both MyD88- and TRIF-dependent signaling, which usually results in serious harmful inflammation *in vivo*. Monophosphoryl lipid A (MPLA), a derivate of LPS, mainly induces TRIF- but not MyD88-dependent signaling, leading to some protective immune responses *in vivo* instead [16,17,18]. Interestingly, TRIM32-deficiency also increased MPLA-induced transcriptions of these genes in BMDMs (Fig 1C). These results suggest that TRIM32 negatively regulates TLR3/4- but not TLR2/7-mediated signaling in primary mouse cells. Consistently, overexpression of TRIM32 markedly inhibited poly(I:C)- and LPS-induced activation of the IFN-β promoter, ISRE and NF-κB in human HEK293-TLR3 and HEK293-TLR4 cells respectively in reporter assays (Fig 1D). In similar experiments, TRIM32 did not inhibit IFNγ-induced activation of the IRF1 promoter (Fig 1E).

### TRIM32 negatively regulates TLR3/4-mediated innate immune and inflammatory responses *in vivo*

**Fig 2:**
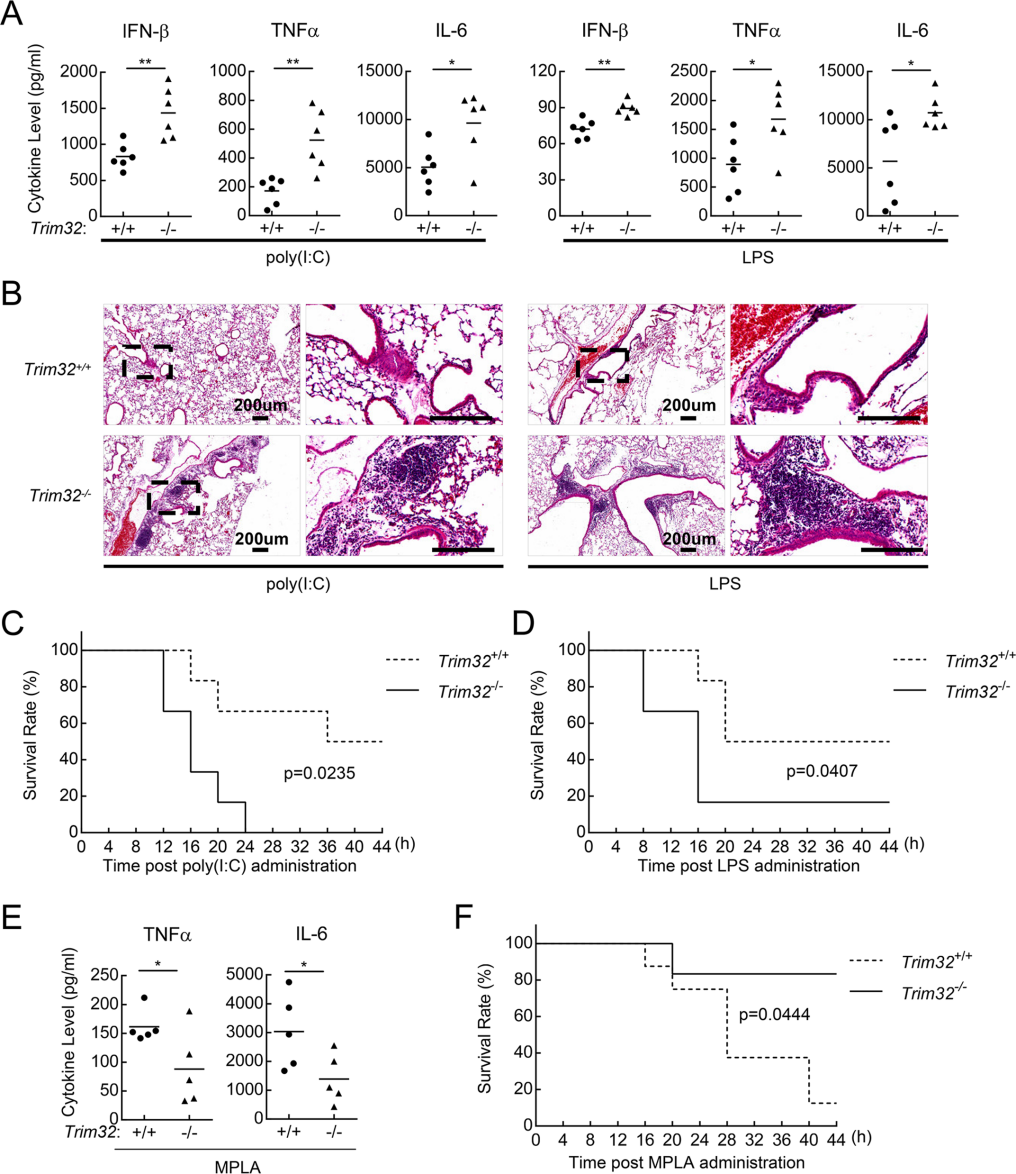
TRIM32-deficiency potentiates TLR3/4-mediated immune responses *in vivo*. (A) Serum cytokine concentrations in *Trim32*^+/+^ and *Trim32*^-/-^ mice. Sex- and age-matched *Trim32*^+/+^ and *Trim32*^-/-^ mice (n = 6) were injected intraperitoneally with poly(I:C) plus D-galactosamine or LPS for the indicated times and the concentrations of IFN-β, TNFα and IL-6 in the serum were determined by ELISA. (B) Effects of TRIM32-deficiency on poly(I:C) and LPS-induced inflammation in the lungs of mice. Sex- and age-matched *Trim32*^+/+^ and *Trim32*^-/-^ mice were injected intraperitoneally with poly(I:C) (2 μg/g) plus D-galactosamine (1 mg/g) or LPS (10 μg /g) for 6 hours and lung sections were used for histological analysis (H&E staining). (C-D) Effects of TRIM32-deficiency on poly(I:C)- and LPS-induced inflammatory death of mice. Wild-type and *Trim32*^−/−^ littermates (n = 6) were treated with poly(I:C) plus D-galactosamine (C) or LPS (D) as in (A). The survival rates of the mice were recorded every 4 hours in the following 44 hours. (E) Serum cytokine concentrations in *Trim32*^+/+^ and *Trim32*^-/-^ mice. Sex- and age-matched *Trim32*^+/+^ and *Trim32*^-/-^ mice (n = 5) were injected intraperitoneally with MPLA (3 mg/g) plus D-galactosamine (1 mg/g) for 2 hours and the concentrations of TNFα and IL-6 in the sera were measured by ELISA. (F) Effects of TRIM32-deficiency on MPLA plus D-galactosamine-induced inflammatory death of mice. Sex- and age-matched *Trim32*^+/+^ (n = 8) and *Trim32*^-/-^ mice (n = 6) were injected intraperitoneally with MPLA (3 mg/g) plus D-galactosamine (1 mg/g). The survival rates of the mice were recorded every 4 hours in the following 44 hours. *, p<0.05; **, p<0.01.

To investigate the role of TRIM32 in TLR3/4-mediated innate immune and inflammatory responses *in vivo*, age- and sex-matched *Trim32*^+/+^ and *Trim32*^−/−^ mice were intraperitoneally injected with poly(I:C) plus D-galactosamine or LPS. D-galactosamine is an agent usually used to enlarge inflammatory damage of liver, since poly(I:C) alone is insufficient to cause inflammatory death of mice. As shown in Fig 2A, poly(I:C)- and LPS-induced production of IFN-β, TNFα, and IL-6 was significantly increased in the sera of *Trim32*^−/−^ compared to *Trim32*^+/+^ mice. Consistently, more serious inflammation was observed in the lungs of *Trim32*^−/−^ mice injected with poly(I:C) plus D-galactosamine or LPS (Fig 2B). *Trim32*^−/−^ mice showed an early death onset and a significantly higher percentage of lethality within 40 hours in comparison with their wild-type counterparts after injection of poly(I:C) plus D-galactosamine (Fig 2C) or LPS (Fig 2D). It has been reported that poly(I:C) and LPS are able to induce TRIF-dependent cell death which might contribute to poly(I:C)- and LPS-induced death of mice [19,20]. Therefore, we also explored whether TRIM32 is involved in TLR3/4-mediated and TRIF-dependent cell necrosis. The results showed that TRIM32-deficiency had no marked effects on poly(I:C)- and LPS-induced cell death in cell viability assays (S2 Fig). We have also explored the role of TRIM32 in MPLA-induced TRIF-dependent protective immune response in mice. Unlike LPS, MPLA does not cause much inflammatory response, and MPLA alone is insufficient to cause inflammatory death of mice. Therefore, we used D-galactosamine to enlarge the inflammatory response induced by MPLA. Interestingly, though MPLA-induced increased transcriptions of type I IFNs and inflammatory genes in *Trim32*^-/-^ cells (Fig 1C), MPLA plus D-galactosamine-induced serum inflammatory cytokine levels and inflammatory death were markedly decreased in *Trim32*^-/-^ mice (Fig 2E & 2F), suggesting that TRIM32 plays an important role in MPLA-induced TRIF-dependent protective immune response *in vivo*.

**Fig 3:**
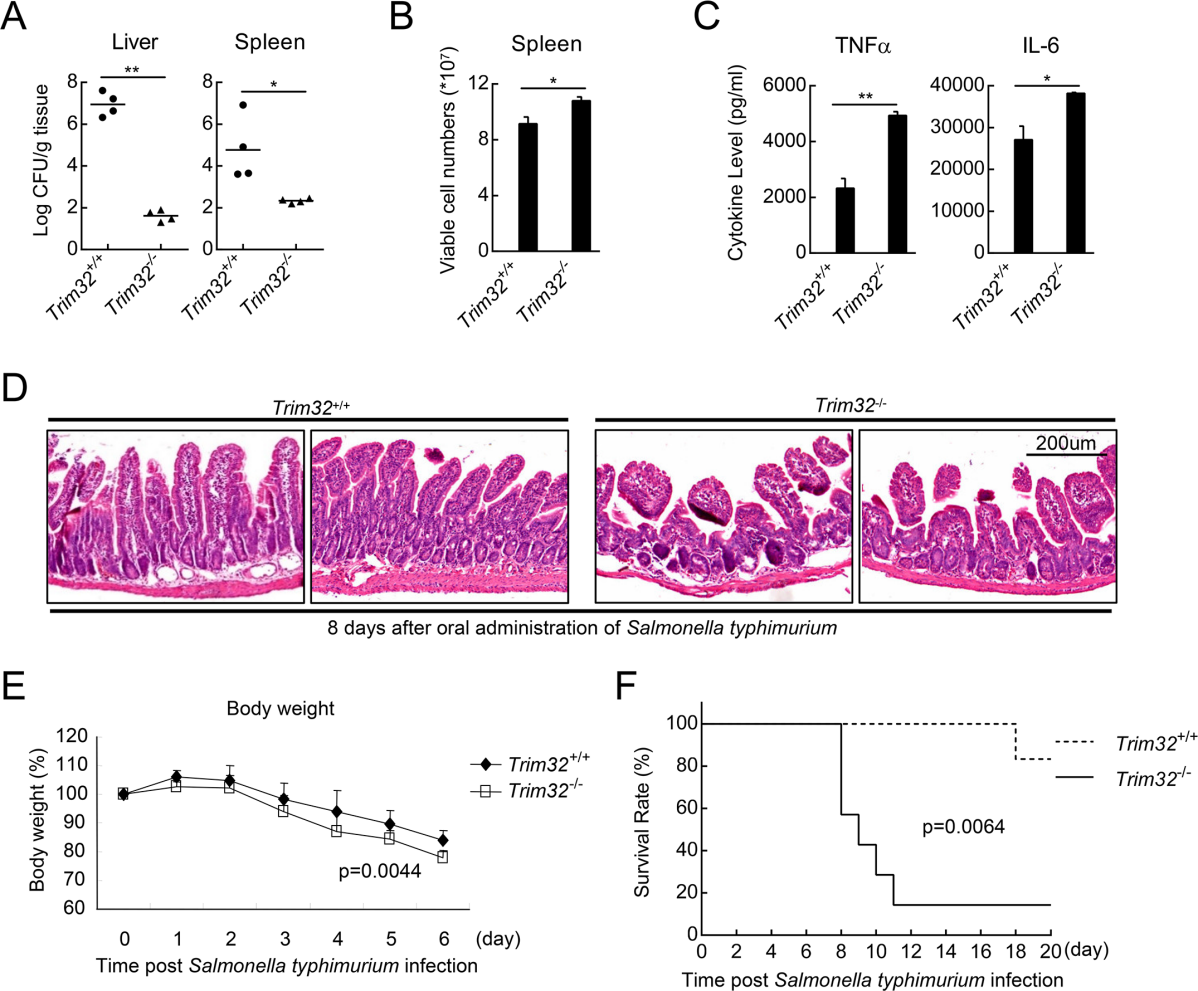
TRIM32-deficiency potentiates *Salmonella typhimurium*-induced immune responses *in vivo*. (A) Effects of TRIM32-deficiency on clearance of *Salmonella typhimurium in vivo*. Sex- and age-matched *Trim32*^*+/+*^ and *Trim32*^*-/-*^ mice were orally infected with *Salmonella typhimurium*, and bacterial loads were assessed in the indicated tissues 8 days post infection. (B) Effects of TRIM32-deficiency on *Salmonella typhimurium*-induced lymphocyte activation. Sex- and age-matched *Trim32*^*+/+*^ and *Trim32*^*-/-*^ mice were orally infected with *Salmonella typhimurium*, and viable cell numbers of spleen were counted 6 days post infection. (C) Effects of TRIM32-deficiency on *Salmonella typhimurium*-induced secretion of inflammatory cytokines. Sex- and age-matched *Trim32*^+/+^ and *Trim32*^-/-^ mice (n = 3) were orally infected with *Salmonella typhimurium* for 6 days, followed by measurement of the levels of inflammatory cytokines in the sera. (D) Effects of TRIM32-deficiency on *Salmonella typhimurium*-induced inflammatory damage of the intestines of mice. Sex- and age-matched *Trim32*^+/+^ and *Trim32*^-/-^ mice were orally infected with *Salmonella typhimurium*, and the intestines of mice were used for histological analysis (H&E staining). (E) Effects of TRIM32-deficiency on *Salmonella typhimurium*-induced loss of body weight. Sex- and age-matched *Trim32*^*+/+*^ (n = 11) and *Trim32*^−/−^ (n = 7) mice were orally infected with *Salmonella typhimurium*, and their body weight were monitored every day for 7 days. (F) Effects of TRIM32-deficiency on *Salmonella typhimurium*-induced inflammatory death. Sex- and *Trim32*^+/+^ (n = 6) and *Trim32*^-/-^ mice (n = 7) were infected with *Salmonella typhimurium*, and the survival rates of the mice were monitored every day for 20 days. *, p<0.05; **, p<0.01.

We have also explored the role of TRIM32 in immune and inflammatory responses to *Salmonella typhimurium* infection. As shown in Fig 3A, *Trim32*^-/-^ mice carried less *Salmonella typhimurium* in their livers and spleens compared with that of their wild-type littermates at 8 days post oral administration of *Salmonella typhimurium*, suggesting that *Trim32*^-/-^ mice exhibited more efficient clearance of invaded *Salmonella typhimurium* than the wild-type mice. Consistently, a larger number of viable immune cells existed in the spleens of *Trim32*^-/-^ mice (Fig 3B). *Trim32*^-/-^ mice produced much higher levels of inflammatory cytokines including TNF-α and IL-6 (Fig 3C) and showed much more serious inflammatory damage of their small intestinal villus (Fig 3D) after oral adiminstration of *Salmonella typhimurium*, which led to a higher sensitivity to *Salmonella typhimurium*-induced loss of body weight and inflammatory death of *Trim32*^-/-^ mice (Fig 3E & 3F). These results suggest that TRIM32 negatively regulates TLR3/4-mediated innate immune and inflammatory responses *in vivo*.

### TRIM32 destablizes TRIF independent of its E3 ligase activity

**Fig 4:**
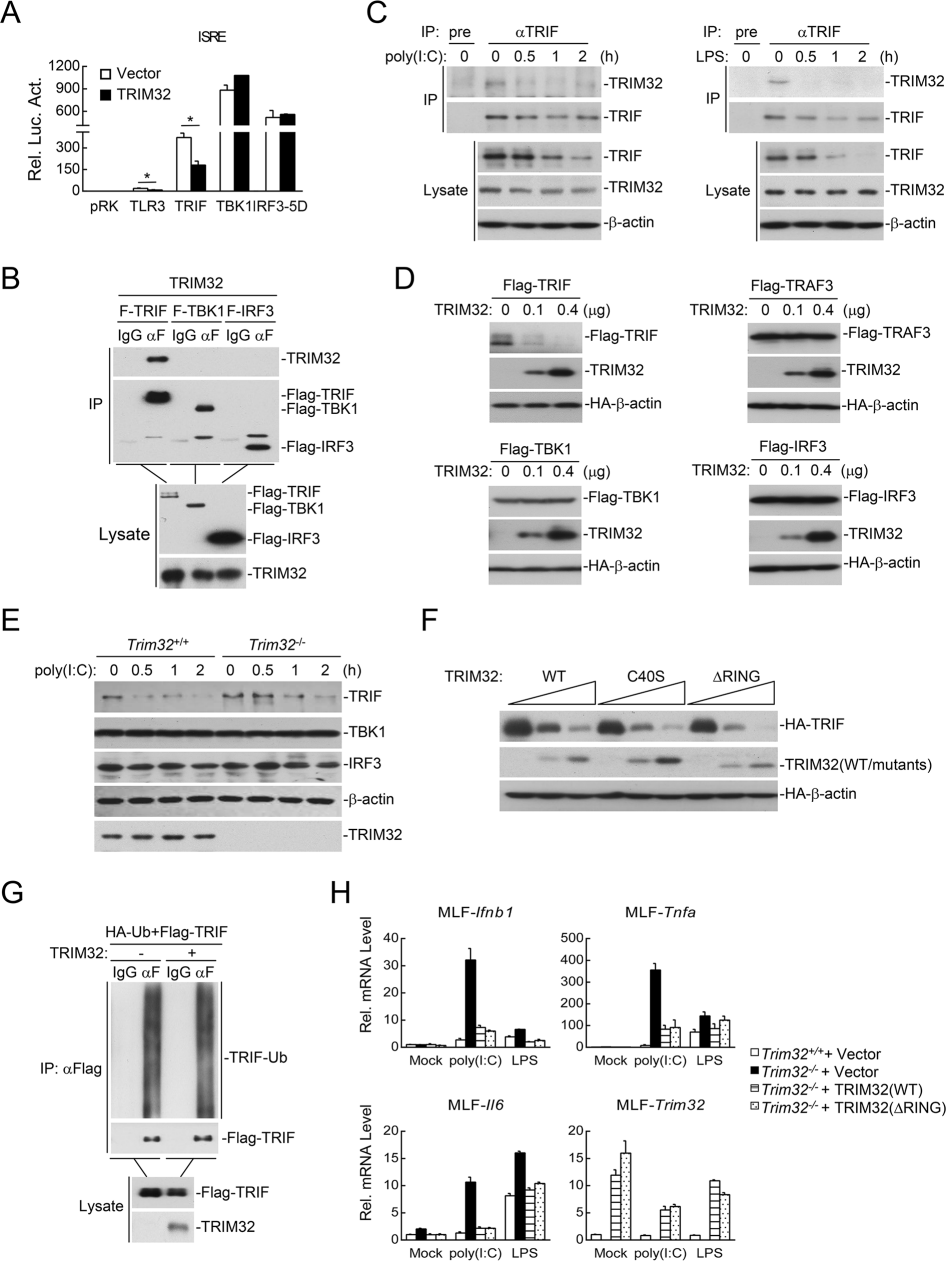
TRIM32 destabilizes TRIF independent of its E3 ligase activity. (A) Murine TRIM32 inhibited TLR3- and TRIF-mediated ISRE activation in mammalian overexpression system. HEK293 cells were transfected with the indicated adaptor plasmid and ISRE reporter (0.1 μg each) and an HA-tagged expression plasmid for murine TRIM32 (0.05 μg). Twenty hours after transfection, luciferase assays were performed. *, p<0.05. (B) Interaction of TRIM32 and TRIF. HEK293 cells were transfected with the indicated plasmids for 24 hours before co-immunoprecipitation and immunoblotting analysis with the indicated antibodies. (C) Endogenous association of TRIM32 with TRIF. MLFs were left untreated or treated with poly(I:C) (100 μg/ml) or LPS (100 ng/ml) for the indicated times, followed by endogenous coimmunoprecipitation and immunoblotting analysis with the indicated antibodies. (D) Effects of murine TRIM32 on levels of TRIF, TRAF3, TBK1 and IRF3. HEK293 cells were transfected with the indicated plasmids for 24 hours before immunoblotting analysis with the indicated antibodies. (E) Effects of TRIM32-deficiency on poly(I:C)-indiced degradation of TRIF. MLFs were left untreated or treated with poly(I:C) (50 μg/ml) for the indicated times before immunoblotting analysis with the indicated antibodies. (F) Effects of murine TRIM32 and its enzyme-inactive mutants on the levels of TRIF. HEK293 cells were transfected with the indicated plasmids for 24 hours before immunoblotting analysis with the indicated antibodies. (G) Effects of murine TRIM32 on ubiquitination of TRIF. HEK293 cells were transfected with the indicated plasmids for twenty hours before immunoprecipitation and immunoblotting analysis with the indicated antibodies. (H) Analysis of poly(I:C)- or LPS-induced transcription of downstream genes in *TRIM32*^-/-^ MLFs reconstituted with an empty vector, TRIM32-Flag (WT) or TRIM32 (ΔRING)-Flag. The reconstituted MLFs were stimulated with poly(I:C) (50 μg/ml) or LPS (50 ng/ml) for the indicated times before qPCR experiments.

We next investigated the molecular mechanisms of TRIM32 in the regulation of TLR3/4-mediated signaling. Reporter assays showed that TRIM32 inhibited TRIF-, but not TBK1- and IRF3-mediated activation of ISRE (Fig 4A). Furthermore, both overexpression and endogenous coimmunoprecipitation experiments indicated that TRIM32 interacted with TRIF (Fig 4B and 4C). In addition, we routinely found that TRIM32 dramatically destabilized TRIF but not TRAF3, TBK1 or IRF3 in our co-transfection experiments (Fig 4D). Endogenous experiments indicated that TRIM32-deficiency markedly attenuated poly(I:C)-induced degradation of TRIF in MLFs (Fig 4E). These results suggest that TRIM32 mediates the down-regulation of TRIF, which is an adaptor protein specifically utilized by TLR3/4 but not other TLRs.

Since TRIM32 is an E3 ubiquitin ligase, we examined whether TRIM32 destabilizes TRIF via the ubiquitin-proteasomal pathway. Unexpectedly, the E3 enzyme-inactive mutants of TRIM32, TRIM32(C40S) and TRIM32(ΔRING), destabilized TRIF as efficient as the wild-type TRIM32 (Fig 4F). Furthermore, TRIM32 failed to catalyze polyubiquitination of TRIF (Fig 4G). Consistently, reconstitution of either wild-type TRIM32 or TRIM32(ΔRING) in *Trim32*^*-/-*^ cells could inhibit poly(I:C)- and LPS-induced transcription of *Ifnb1*, *Tnfa* and *Il6* genes to similar levels (Fig 4H). These data suggest that TRIM32 mediates the down-regulation of TRIF independent of its E3 ligase activity.

### TRIM32 mediates autophagic degradation of TRIF

**Fig 5:**
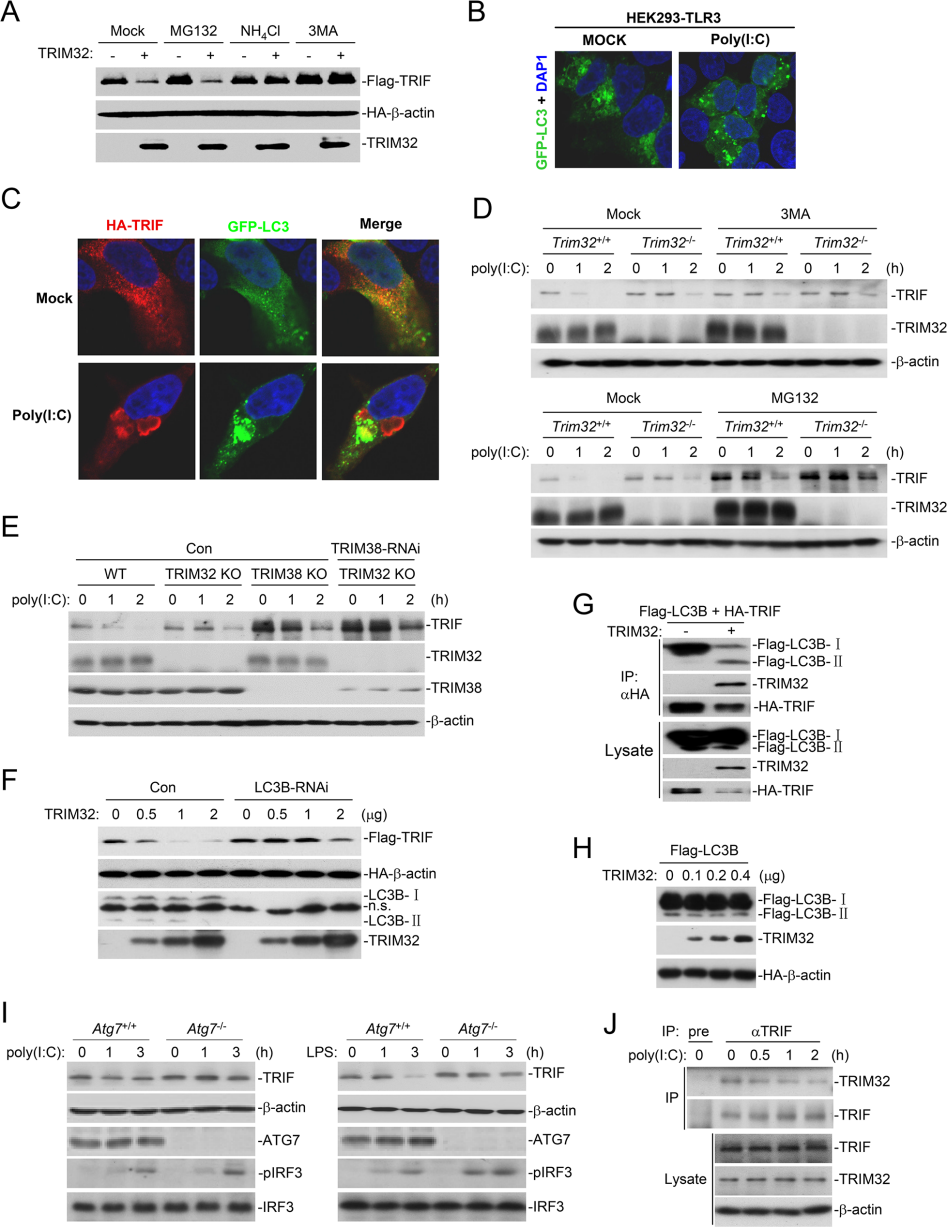
TRIM32 promotes TAX1BP1-mediated selective autophagic degradation of TRIF. (A) Effects of different inhibitors on TRIM32-mediated destabilization of TRIF. HEK293 cells were transfected with the indicated plasmids for 20 hours and then treated with the indicated inhibitors for 6 hours before immunoblotting analysis with the indicated antibodies. (B) Effects of poly(I:C) treatment on autophagy. HEK293-TLR3 cells were transfected with a GFP-tagged expression plasmid for LC3. Twenty hours after transfection, cells were treated with poly(I:C) (50 ng/mL) for 3 hours and subjected for confocal microscopy. (C) Effects of poly(I:C) treatment on colocalization of TRIF and LC3. HEK293-TLR3 cells were transfected with the indicated plasmids. Twenty hours after transfection, cells were treated with poly(I:C) (50 ng/mL) for 3 hours before subjected for confocal microscopy. (D) Effects of 3MA and MG132 on poly(I:C)-induced degradation of TRIF. *Trim32*^+/+^ and *Trim32*^-/-^ BMDMs were pre-treated with 3MA or MG132 for 2 hours, followed by poly(I:C) stimulation for the indicated times before immunoblotting analysis was performed. (E) Effects of double deficient of TRIM32 and TRIM38 on poly(I:C)-induced degradation of TRIF. *Trim32*^+/+^ and *Trim32*^-/-^ MLFs stably transduced with control or TRIM38-RNAi plasmids were left untreated or treated with poly(I:C) (50 μg/ml) for the indicated times before immunoblotting analysis with the indicated antibodies. (F) Knockdown of LC3B inhibits TRIM32-mediated degradation of TRIF. HEK293 cells were transfected with a RNAi control or LC3B-RNAi plasmid for 24 hours, and then further transfected with the indicated plasmids for 24 hours before immunoblotting analysis with the indicated antibodies. (G) Effects of TRIM32 on interaction of TRIF with LC3B. HEK293 cells were transfected with the indicated plasmids for 24 hours before coimmunoprecipitation and immunoblotting analysis with the indicated antibodies. (H) Effects of TRIM32 on the conversion of LC3B-I to LC3B-II. HEK293 cells were transfected with the indicated plasmids for 24 hours before coimmunoprecipitation and immunoblotting analysis with the indicated antibodies. (I) Effects of ATG7-deficiency on poly(I:C)- or LPS-induced degradation of TRIF. *Atg7*^+/+^ and *Atg7*^-/-^ MEFs (2x10^6^) were treated with poly(I:C) (50 μg/ml) or LPS (50 ng/ml) for the indicated times before immunoblotting analysis with the indicated antibodies. (J) Endogenous association of TRIM32 with TRIF. MLFs were pre-treated with bafilomycin for 2 hours, followed by poly(I:C) stimulation for the indicated times before coimmunoprecipitation was performed.

Protein degradation is one of the main strategies involved in inactivating proteins in biological processes. Two major systems exist for protein degradation, including the ubiquitin-proteasome and autophagy-lysosome pathways. We found that TRIM32-mediated degradation of TRIF could be inhibited by the lysosomal inhibitor NH_4_Cl and the autophagic inhibitor 3MA but not the proteasomal inhibitor MG132 (Fig 5A), suggesting that TRIM32 probably mediates degradation of TRIF via an autophagic pathway. Confocal microscopy experiments showed that poly(I:C) stimulation caused aggregation of GFP-LC3 (a marker of autophagy) in HEK293-TLR3 cells (Fig 5B). Poly(I:C) stimulation also caused colocalization of TRIF with GFP-LC3 in HEK293-TLR3 cells (Fig 5C). To further confirm that autophagic degradation pathway is involved in TRIF degradation upon stimulation, we used MG132 and 3MA to pre-treat *Trim32*^+/+^ and *Trim32*^-/-^ BMDMs for 2 hours before poly(I:C) stimulation. The results showed that 3MA pre-treatment had no marked effects on TRIF level in both un-stimulated *Trim32*^+/+^ and *Trim32*^-/-^ cells, but attenuated poly(I:C)-induced degradation of TRIF in *Trim32*^+/+^ but not *Trim32*^-/-^ cells (Fig 5D). MG132 pre-treatment markedly increased TRIF level in un-stimulated cells and also attenuated poly(I:C)-induced degradation of TRIF in both *Trim32*^+/+^ and *Trim32*^-/-^ cells (Fig 5D). TRIF has been reported to be degraded by TRIM38 via the ubiquitin-proteasome dependent pathway, and TRIM38-deficiency increases TRIF level in un-stimulated cells and attenuates poly(I:C)-induced degradation of TRIF [21]. Consistently, knockdown of TRIM38 increased TRIF level in un-stimulated *Trim32*^-/-^ cells, and also attenuated poly(I:C)-induced degradation of TRIF to a larger extent in these cells (Fig 5E). Taken together, these results suggest that TRIM32 is involved in the autophagic degradation of TRIF induced by poly(I:C) stimulation. Additional experiments showed that knockdown of LC3B, which is an important marker of autophagy and is required for fusion to the lysosomes, markedly inhibited TRIM32-mediated degradation of TRIF (Fig 5F), whereas overexpression of TRIM32 dramatically enhanced the interaction of TRIF with LC3B-II (Fig 5G), which is a basic membrane component of autophagesomes and derived from LC3B-I during autophagy [22]. In similar experiments, overexpression of TRIM32 did not cause the conversion of LC3B-I to LC3B-II (Fig 5H). Moreover, deficiency of ATG7, which is an essential E1 ligase for LC3B-II formation, inhibited poly(I:C)- and LPS-induced degradation of TRIF (Fig 5I). These results suggest that TRIM32 mediates degradation of TRIF through the autophagy-lysosome pathway. Consistently, pre-treatment of cells with balifomycin, an inhibitor for fusion of autophagosomes and lysosomes, markedly attenuated poly(I:C)-induced down-regulation of TRIF (Fig 5J). Endogenous TRIM32 constitutively associated with TRIF in un-stimulated cells, and their association slowly decreased following poly(I:C) stimulation (Fig 5J), suggesting that TRIM32 disassociates from TRIF-containing autophagosomes at the later stage of stimulation.

### TRIF is degraded by TRIM32-TAX1BP1-mediated selective autophagy

**Fig 6:**
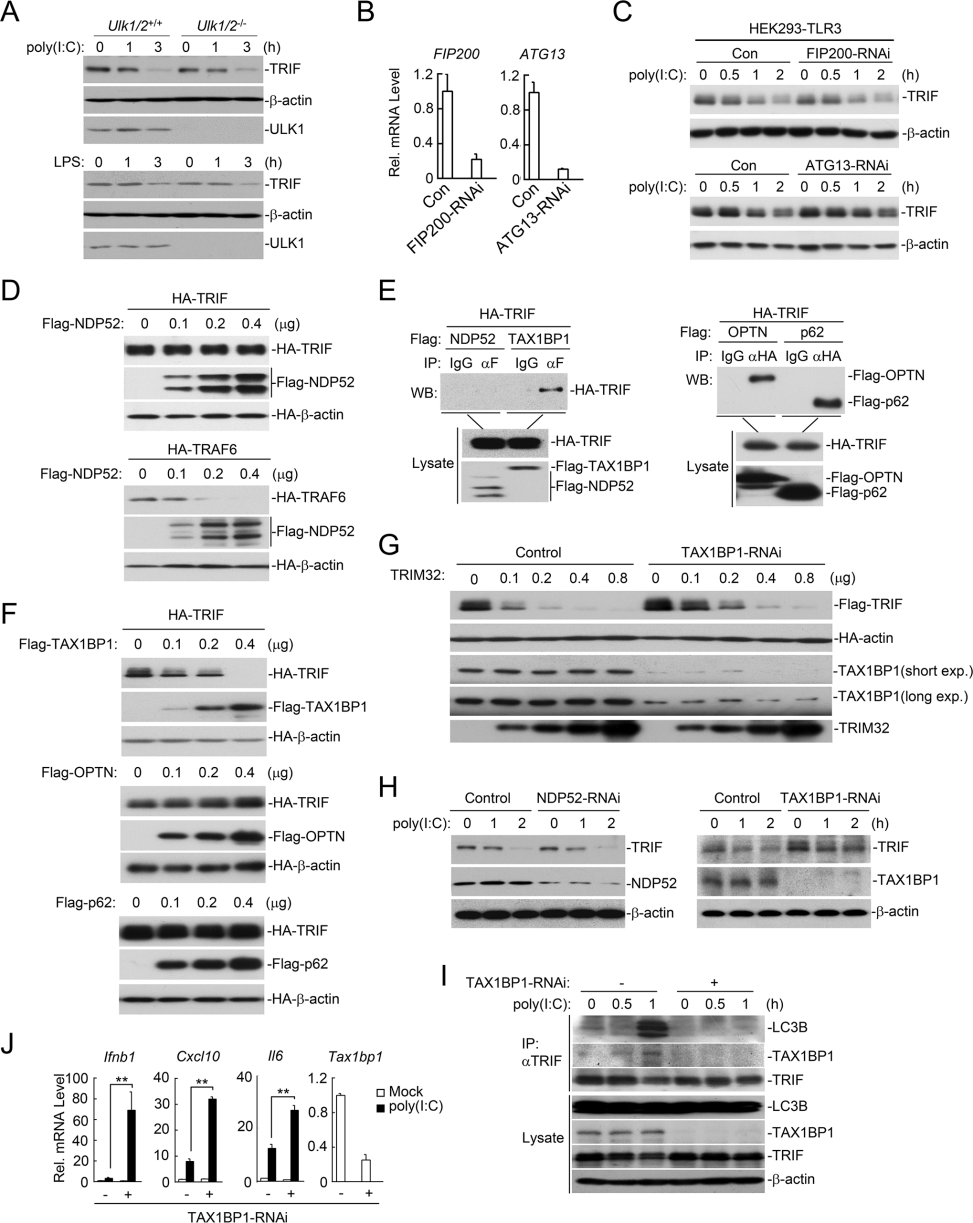
TRIM32 promotes TAX1BP1-mediated selective autophagic degradation of TRIF. (A) Effects of ULK1/2-deficiency on poly(I:C)- or LPS-induced degradation of TRIF. *Ulk1/2*^+/+^ and *Ulk1/2*^-/-^ MEFs were left untreated or treated with poly(I:C) (50 μg/ml) or LPS (50 ng/ml) for the indicated times before immunoblotting analysis with the indicated antibodies. (B) Examination of the knockdown efficiency of FIP200-RNAi and ATG13-RNAi. HEK293-TLR3 cells (4 × 10^5^) were transfected with the indicated RNAi plasmids (1 μg each) for 36 hours before qPCR was performed. (C) Effects of FIP200-RNAi and ATG13-RNAi on poly(I:C)-induced degradation of TRIF. HEK293-TLR3 cells (4 × 10^5^) were transfected with the indicated RNAi plasmids (1 μg each) for 36 hours and then further treated with poly(I:C) (50 μg/ml) for the indicated times before immunoblotting analysis with the indicated antibodies. (D) Effects of NDP52 on levels of TRIF and TRAF6. HEK293 cells were transfected with the indicated plasmids before immunoblotting analysis with the indicated antibodies. (E) Interaction of TRIF with NDP52, TAX1BP1, OPTN and p62 in mammalian overexpression system. HEK293 cells were transfected with the indicated plasmids for 24 hours before communoprecipitation and immunoblotting analysis with the indicated antibodies. (F) Effects of TAX1BP1, OPTN and p62 on expression of TRIF. HEK293 cells were transfected with the indicated plasmids for 24 hours before immunoblotting analysis with the indicated antibodies. (G) Effects of TAX1BP1-RNAi on TRIM32-mediated degradation of TRIF. The control and TAX1BP1-RNAi stably transduced cells were transfected with the indicated plasmids for 24 hours before immunoblotting analysis with the indicated antibodies. (H) Effects of NDP52-RNAi or TAX1BP1-RNAi on poly(I:C)-induced degradation of TRIF. MLFs stably transduced with the indicated RNAi plasmids were left untreated or treated with poly(I:C) (50 μg/ml) for the indicated times before immunoblotting analysis with the indicated antibodies. (I) Effects of TAX1BP1-RNAi on poly(I:C)-induced association of TRIF with LC3B. MLFs stably transduced with the indicated RNAi plasmids were left un-treated or treated with poly(I:C) (50 μg/ml) for the indicated times before coimmunoprecipitation was performed. (J) Effects of TAX1BP1-RNAi on poly(I:C)-induced transcription of *Ifnb1*, *Cxcl10* and *IL-6* genes. MLFs stably transduced with the indicated RNAi plasmids were left untreated or treated with poly(I:C) (50 μg/ml) for 2 hours before qPCR analysis. **, p<0.01.

The autophagic pathways can be distinguished as the canonical or the selective autophagic pathway. The selective autophagy receptors deliver cargoes to the autophagosomes for selective degradation [23]. It has been shown that ULK1/2, FIP200 and ATG13 are critical for initiation of the classical autophagic pathway [23]. We found that deficiency of ULK1/2, FIP200 or ATG13 had no marked effects on the degradation of TRIF induced by poly(I:C) or LPS treatment (Fig 6A–6C), suggesting that poly(I:C)- and LPS-induced degradation of TRIF is not via the canonical autophagic pathway. It has been shown that NDP52 serves as a selective receptor for TRIF and TRAF6 for their selective autophagic degradation [24]. However, in our experiments, we observed that NDP52 failed to interact with and promote degradation of TRIF (Fig 6D & 6E). In similar experiments, NDP52 promoted the degradation of TRAF6 (Fig 6D). Instead, we found that TRIF interacted with other selective receptors including TAX1BP1, OPTN and p62 (Fig 6E), but only overexpression of TAX1BP1, but not OPTN or p62 down-regulated the level of TRIF (Fig 6F). Consistently, knockdown of TAX1BP1 inhibited TRIM32-mediated degradation of TRIF (Fig 6G). In addition, knockdown of TAX1BP1 but not NDP52 attenuated poly(I:C)-induced degradation of TRIF (Fig 6H). Furthermore, knockdown of TAX1BP1 markedly impaired endogenous association of TRIF with LC3 induced by poly(I:C) stimulation (Fig 6I). These results suggest that TAX1BP1 but not NDP52 mediates the selective autophagic degradation of TRIF. Consistent with the biochemical results, qPCR experiments showed that knockdown of TAX1BP1 potentiated poly(I:C)-induced transcription of *Ifnb1*, *Cxcl10* and *Il6* genes (Fig 6J).

**Fig 7:**
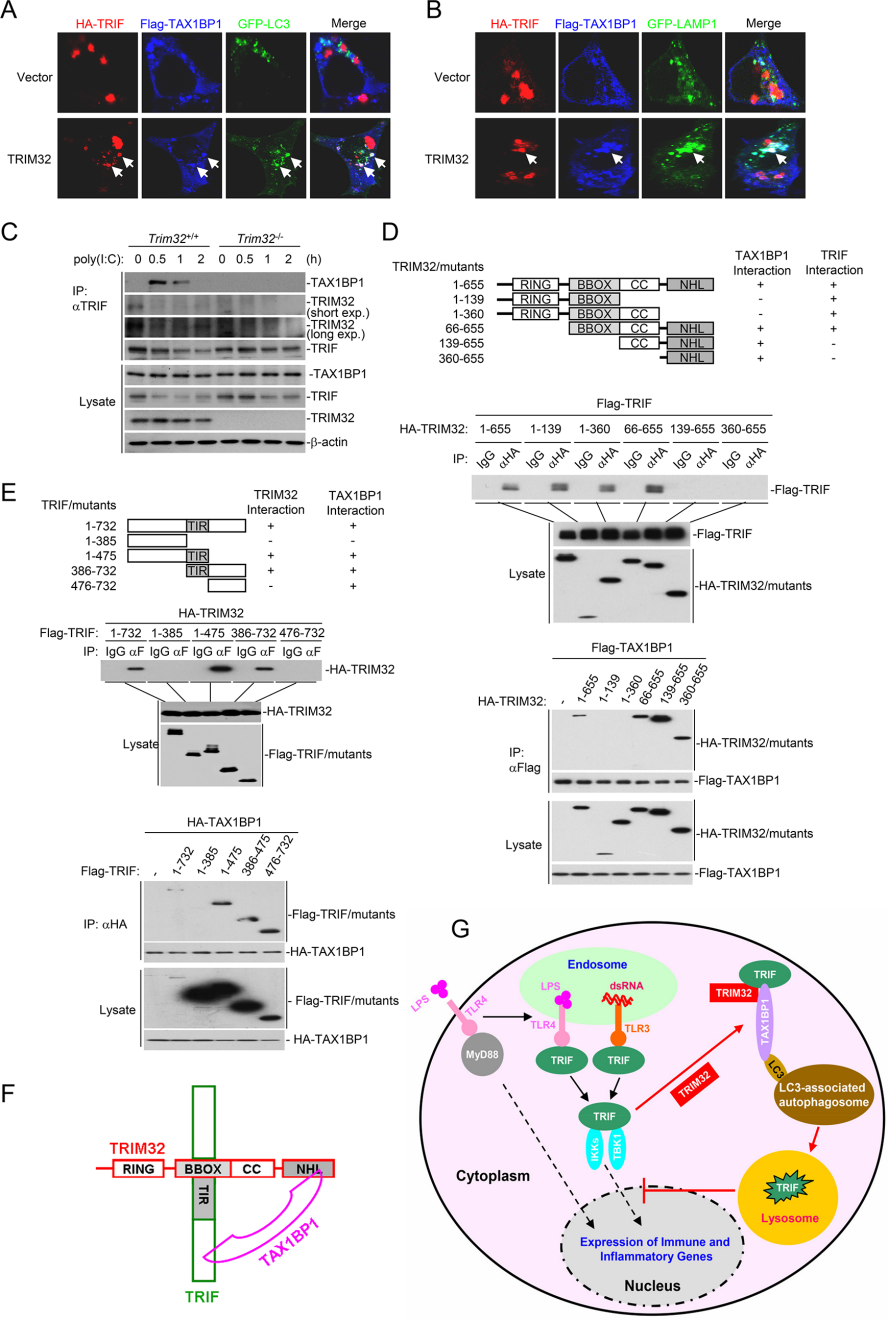
TRIM32 links TRIF and TAX1BP1 through distinct domains. (A-B) Effects of TRIM32 on colocalization of TRIF, TAX1BP1 and LC3 or LAMP1. HEK293 cells were transfected with the indicated plasmids for 20 hours, and then were fixed with 4% paraformaldehyde and subjected for confocal microscopy. (C) Effects of TRIM32-deficiency on poly(I:C)-induced TRIF-TAX1BP1 association. *TRIM32*^+/+^ and *TRIM32*^-/-^ MLFs were left untreated or treated with poly(I:C) (50 μg/mL) for the indicated times before coimmunoprecipitation and immunoblotting analysis with the indicated antibodies. (D) Interactions of TRIM32 mutants with TAX1BP1 and TRIF. HEK293 cells were transfected with the indicated plasmids before coimmunoprecipitation and immunoblotting analysis with the indicated antibodies. (E) Interactions of TRIF mutants with TRIM32 and TAX1BP1. The experiments were similar performed as in (D). (F) A schematic presentation of interactions among TRIF, TRIM32 and TAX1BP1. (G) A model on the role of TRIM32 in regulation of TLR3/4-mediated signaling.

To explore the mechanism of TRIM32- and TAX1BP1-mediated autophagic degradation of TRIF, we tested a straightforward hypothesis that TRIM32 acts as a bridge protein for TRIF-TAX1BP1 interaction. Confocal microscopy indicated that overexperssion of TRIM32 promoted colocolization of TRIF and TAX1BP1 in certain aggregates that were positive for the autophagosome marker GFP-LC3 (Fig 7A) or lysosome marker GFP-LAMP1 (Fig 7B). Endogenous coimmunoprecipitation experiments indicated that TRIM32-deficiency abolished poly(I:C)-induced association of TRIF with TAX1BP1 as well as attenuated poly(I:C)-induced degradation of TRIF (Fig 7C). These results suggest that TRIM32 acts as a bridge protein for TRIF-TAX1BP1 interaction following poly(I:C) stimulation.

Domain mapping experiments indicated that the NHL (aa360-655) and BBOX (aa66-139) domains of TRIM32 are required for its interaction with TAX1BP1 and TRIF respectively (Fig 7D). Similar experiments indicated that TRIM32 interacted with the middle TIR domain (aa386-475) of TRIF (Fig 7E), whereas TAX1BP1 interacted most strongely with the C-terminal domain (aa476-732) of TRIF (Fig 7E). These results suggest that TRIM32 links TAX1BP1 and TRIF through distinct domains (Fig 7F).

Additionally, we have also explored whether TRIM32 and TAX1BP1 are recruited to lipid rafts of membrane where TLR3/4 recruits TRIF for signaling. Cellular fractionation experiments indicated that membrane-associated TRIF was increased at 0.5 hour after poly(I:C) treatment and then decreased at 1 hour probably because of the degradation of TRIF (S3 Fig). TRIM32 constitutively existed in both cytosol and membrane franction, and poly(I:C) treatment had no marked effects on its distribution. Interestingly, TAX1BP1 only existed in the cytosol either before or after poly(I:C) treatment. Furthermore, TRIF-deficiency had no marked effects on the subcellular location of TRIM32 and TAX1BP1 either before or after poly(I:C) treatment (S3 Fig). These results suggest that TAX1BP1 is not recruited to lipid rafts of membrane, and TRIM32-TAX1BP1-TRIF association occurs in the cytosol after TRIF is dis-associated from the TLR3/4 receptor complexes on the membrane.

## Discussion

In this study, we investigated the role of TRIM32 in TLR3/4-mediated signaling in mouse primary immune cells and *in vivo* by genetic and biochemical studies. TRIM32-deficiency potentiated poly(I:C)- and LPS- but not R848- or PGN-induced transcription of downstream genes *Ifnb1*, *Isg56*, *Tnfa* and *Il6* in BMDMs, BMDCs and MLFs. TRIM32-deficiency also elevated the serum cytokine levels induced by poly(I:C) and LPS, and renders the mice more susceptible to death triggered by administration of poly(I:C) and LPS or *Salmonella typhimurium* infection. These findings suggest that TRIM32 negatively regulates TLR3/4-mediated innate immune and inflammatory responses.

It has been shown that TRIM32 is an E3 ubiquitin ligase which regulates both DNA- and RNA viruses-triggered induction of type I IFNs in several human cell lines [7]. The current study indicates that TRIM32 is not required for induction of downstream antiviral genes induced by both DNA and RNA viruses in primary mouse cells or in mice. It is possible that TRIM32 functions in different cellular processes between human and mouse cells. TRIM proteins belong to the largest E3 ubiquitin ligase family in mammals, and it has been previously shown that some TRIM family members have distinct functions between human and mouse [25]. In contrast with the observations that the E3 ligase activity of TRIM32 is required for its roles in virus-triggered signaling in human cell lines [7], several results from the current study suggest that the E3 ligase activity of murine TRIM32 is not required for its negative regulatory roles in TLR3/4-mediated signaling. Firstly, the E3 enzyme-inactive mutants of TRIM32 destabilized TRIF as efficiently as the wild-type protein. Second, reconstitution of both the wild-type and E3 enzyme-inactive TRIM32 into *Trim32*^-/-^ cells inhibited the transcription of downstream genes induced by poly(I:C) and LPS. TRIF is a critical adaptor protein for TLR3/4-mediated innate immune and inflammatory responses. Poly(I:C) or LPS stimulation causes a rapid and dramatic degradation of TRIF to avoid sustained activation of TRIF and expression of type I IFNs and inflammatory cytokines. Previous studies demonstrate that the E3 ubiquitin ligases WWP2 and TRIM38 target TRIF for degradation and inhibit TLR3/4-mediated innate immune responses [21,26]. Both WWP2 and TRIM38 catalyze K48-linked polyubiquitination of TRIF and promote TRIF degradation via the well-established ubiquitin-proteasome system. Instead, TRIM32 promotes TRIF degradation via the autophagic pathway, since the autophagy inhibitor 3MA and lysosome inhibitor NH_4_Cl but not the ubiquitin-proteasome inhibitor MG132 impaired TRIM32-mediated degradation of TRIF. In addition, TRIM32 promoted the interaction of TRIF with LC3B-II, which is the critical component for autophagosome formation. Interestingly, TRIM32 promotes TRIF degradation via the selective instead of the classical autophagic pathway, since deficiency of components of the selective but not classical autophagic pathway inhibited TRIM32-mediated, as well as poly(I:C)- and LPS-induced degradation of TRIF. Our results also indicated that TRIM38 but not TRIM32 down-regulated TRIF level in un-stimulated cells, whereas TRIM32 contributed to ligand-induced degradation of TRIF. Therefore, TRIM38 and TRIM32 regulate TRIF-mediated signaling through distinct mechanisms.

Several experiments suggest that the selective autophagic receptor TAX1BP1 but not NDP52 is involved in TRIM32-mediated autophagic degradation of TRIF. TAX1BP1 but not NDP52 interacted with TRIF. Overexpression of TAX1BP1 but not NDP52 promoted degradation of TRIF, whereas knockdown of TAX1BP1 but not NDP52 impaired TRIM32-mediated as well as poly(I:C)-induced degradation of TRIF. Furthermore, knockdown of TAX1BP1 markedly impaired poly(I:C)-induced endogenous association of TRIF with LC3.

Our experiments suggest that TRIM32 acts as a link for TRIF and TAX1BP1. Confocal microscopy showed that TRIM32 promoted colocalization of TRIF and TAX1BP1 in certain aggregates which are positive for the autophagosome marker GFP-LC3 or the lysosome marker GFP-LAMP1, while TRIM32-deficiency abolished endogenous association of TRIF with TAX1BP1 induced by poly(I:C). Domain mapping experiments indicated that the BBOX and NHL domains of TRIM32 were required for its interaction with the TIR domain of TRIF and TAX1BP1 respectively, whereas TAX1BP1 interacted with the C-terminal domain of TRIF. These results suggest that TRIM32 links TRIF to the TAX1BP1 autophagosomes through distinct domains.

Based on our data, we propose a working model on the regulatory role of TRIM32 in TLR3/4-mediated innate immune responses (Fig 7G). Ligand binding to TLR3/4 leads to the recruitment of the critical adaptor protein TRIF. TRIF in turn recruits downstream components, leading to activation of several transcription factors and ultimate induction of downstream innate immune and inflammatory genes. Upon activation, TRIF is recruited by TRIM32 to TAX1BP1-containing and LC3-associated autophagosomes for degradation, contributing to termination of TLR3/4-mediated innate immune and inflammatory responses. Our findings suggest that selective autophagic degradation is an important regulatory mechanism for timely termination of innate immune and inflammatory responses mediated by TLR3/4.

## Materials and methods

### Ethics statement

All animal experiments were performed in accordance with the Wuhan University animal care and use committee guidelines.

### Reagents and antibodies

Mouse monoclonal antibodies against Flag (Sigma), HA (Origene), β-actin (Sigma), p-IκBα (CST), p-IRF3 (CST) and p-TBK1 (Abcam); poly(I:C) (Invivogen), LPS (Sigma), R848 (Invivogen), PGN (Invivogen), human IFN-γ (PeproTech), Bafilomycin (Sigma), Monophosphoryl lipid A (Sigma), Z-KAD-FMK (MCE) were purchased from the indicated companies. Luminescent cell viability assay kit (G7570) was purchased from Promega. Mouse antisera to TRIM32 and TRIF were raised against recombinant human TRIM32 and murine TRIF(1–475) respectively. Rabbit antisera to TAX1BP1 and NDP52 were raised against recombinant murine TAX1BP1 and NDP52(1–160) respectively.

### Constructs

Mammalian expression plasmids for Flag- or HA-tagged murine TRIM32 and its mutants, TRIF and its mutants, TRAF6, TRAF3, TBK1, IRF3, TAX1BP1 and NDP52 were constructed by standard molecular biology techniques.

### Mice and genotyping

*Trim32* gene knockout mice with a CL7/B6 background were provided by Dr. Hong-Liang Li [27]. Genotyping by PCR was performed using the following two pairs of primers: WT-1: GGAGAGACACTATTTCCTAAGTCA;WT-2: GTTCAGGTGAGAAGCTGCTGCA; MT: GGGACAGGATAAGTATGACATCA.

Amplification of the wild-type allele with primers WT-1 and WT-2 results in a 250-bp fragment, whereas amplification of the disrupted allele with primers WT-1 and MT results in a 300-bp fragment.

### Generation of BMDMs and BMDCs

BMDMs and BMDCs were generated as described [28]. The bone marrow cells (1×10^7^) were cultured in RPMI medium 1640 containing 10% FBS and 10 ng/mL recombinant murine M-CSF (Peprotech) or GM-CSF-containing conditional medium in a 100-mm dish for 5 or 9 days for generation of BMDMs or BMDCs respectively.

### Isolation of MLFs

Primary lung fibroblasts were generated as described [29]. Primary lung fibroblasts were isolated from approximately 4- to 6-week-old mice. Lungs were minced and digested in calcium and magnesium free HBSS containing 10 μg/ml type II collagenase (Worthington) and 20 μg/ml DNase I (Sigma-Aldrich) for 3 hours at 37°C with shaking. Cell suspensions were filtered through progressively smaller cell strainers (100 and 40 μm) and then centrifuged at 1500 rpm for 4 min. The cells were then plated in culture medium (1:1 [v/v] DMEM/Ham’s F-12 containing 10% FBS, 15 mM HEPES, 2 mM L-glutamine, 50 U/ml penicillin, and 50 μg/ml streptomycin). After 1 hour, adherent fibroblasts were rinsed with HBSS and cultured in media.

### Mouse poly(I:C) and LPS injection

Age- and sex-matched *Trim32*^+/+^ and *Trim32*^-/-^ mice were injected intraperitoneally with poly(I:C) (5 μg/g body weight) plus D-galactosamine (1 mg/g body weight) or with LPS (10 μg/g body weight). The survival of the injected mice was monitored every 2 hours.

### Mouse infection with *Salmonella typhimurium*

Age- and sex-matched *Trim32*^+/+^ and *Trim32*^-/-^ mice were orally administrated with *Salmonella typhimurium* (1×10^7^ pfu per mouse). The body weight and survival of the infected mice were monitored every day.

### Measurement of cytokines

Blood from mice injected with poly(I:C) plus D-galactosamine or LPS was collected at the indicated times and the serum concentration of TNFα (Biolegend), IL-6 (Biolegend), and IFN-β (PBL) were measured by ELISA kits from the indicated manufactures.

### Transfection and reporter assays

Transfection and reporter assays were performed as previous described [30,31,32,33]. HEK293 cells were seeded on 24-well plates and transfected on the following day by standard calcium phosphate precipitation. Where necessary, empty control plasmid was added to ensure that each transfection receives the same amount of total DNA. To normalize for transfection efficiency, pRL-TK (*Renilla* luciferase) reporter plasmid (0.01 μg) was added to each transfection. Luciferase assays were performed using a dual-specific luciferase assay kit (Promega, Madison, WI). Firefly luciferase activities were normalized based on *Renilla* luciferase activities.

### Coimmunoprecipitation, immunoblotting and ubiquitination assays

Coimmunoprecipitation, immunoblotting and ubiquitination assays were performed as previous described [34,35,36]. For ubiquitination assays, the immunoprecipitates were re-extracted in lysis buffer containing 1% SDS and denatured by heating for 5 min. The supernatants were diluted with regular lysis buffer until the concentration of SDS was decreased to 0.1%, followed by re-immunoprecipitation with the indicated antibodies. The immunoprecipitates were analyzed by immunoblotting with the ubiquitin antibody.

### qPCR

qPCR assays were performed as previously describe [37,38,39,40]. Total RNA from mouse or human cells was isolated using the Trizol reagent (Invitrogen). After reverse-transcription with oligo(dT) primer using a RevertAidTM First Strand cDNA Synthesis Kit (Fermentas), aliquots of products were subjected to qPCR analysis to measure mRNA levels of the tested genes. *Gapdh* was used as a reference gene. Gene-specific primer sequences were previously described [33,41].

### Immunohistochemistry analysis

Lungs or intestines from mice were fixed in formalin and embedded into paraffin blocks. The paraffin blocks were sectioned (5 μm) for H&E staining. The immunohistochemistry analysis was performed on the 5-μm sections. The sections were placed on polylysinecoated slides, deparaffinized in xylene, rehydrated through graded ethanol, quenched for endogenous peroxidase activity in 3% hydrogen peroxide, and processed for antigen retrieval by microwave heating for 7 min in 10 mM citrate buffer (pH 6.0). Sections were counterstained with hematoxylin (Zymed Laboratories) for 5 min and coverslipped. Pictures were acquired using a HistoFAXS system.

### Subcellular fractionation

*TRIF*^*+/+*^ and *TRIF*^*-/-*^ HEK293-TLR3 cells (5×10^7^) were treated with poly(I:C) for the indicated times, and then cells were harvested and lysed by douncing for 20 times in 2 ml homogenization buffer (10 mM Tris-HCl [pH 7.4], 2 mM MgCl2, 10 mM KCl, and 250 mM sucrose). The homogenate was centrifuged at 500 g for 10 min for removal of the crude nuclei. The supernatant (S5) was centrifuged at 100, 000 g for 2 hours for cytosol (S100K) and membrane (P100K) generation.

### Statistical analysis

Differences between averages were analyzed by Student’s *t*-test. *P* value of less than 0.05 was considered significant.

## Supporting information

S1 Fig TRIM32-deficiency has no markedly effects on virus-triggered induction of downstream antiviral genes in primary mouse cells. (A) PCR genotyping of *Trim32* gene knockout mice. (B) Detection of TRIM32 in *Trim32*^*+/+*^ *and Trim32*^*-/-*^ cells. *Trim32*^*+/+*^ *and Trim32*^*-/-*^ cells were lysed followed by immunoblotting analysis with the indicated antibodies. (C) Effects of TRIM32-deficiency on SeV- and HSV-1-induced transcription of *Ifnb1*, *Isg56*, *Tnfa* and *Il6* in MEFs, BMDMs and BMDCs. Wild-type and *Trim32*^-/-^ cells were infected with SeV or HSV-1 before qPCR analysis.
(TIF)

S2 Fig TRIM32-deficiency has no marked effects on poly(I:C)- and LPS-induced cell death. *Trim32*^*+/+*^ and *Trim32*^*-/-*^ BMDMs were treated with DMSO, z-VAD (10 μM), poly(I:C) (50 μg/mL), poly(I:C) (50 μg/mL) plus z-VAD (10 μM), LPS (20 ng/mL) or LPS (20 ng/mL) plus z-VAD (10 μM) for 24 hours before cell viability assays.
(TIF)

S3 Fig Analysis of the subcellular locations of TRIM32 and TAX1BP1 after poly(I:C) stimulation. *TRIF*^*+/+*^ and *TRIF*^*-/-*^ HEK293-TLR3 cells were treated with poly(I:C) (50 ng/mL) for the indicated times before cell fractionation and immunoblotting analysis with the indicated antibodies.
(TIF)
