# Supplementary figures and images for: TRIM32-TAX1BP1-dependent selective autophagic degradation of TRIF negatively regulates TLR3/4-mediated innate immune responses
Source: PLoS Pathog. 2017 Sep 12;13(9):e1006600. doi: 10.1371/journal.ppat.1006600 (PMC5595311; doi:10.1371/journal.ppat.1006600)

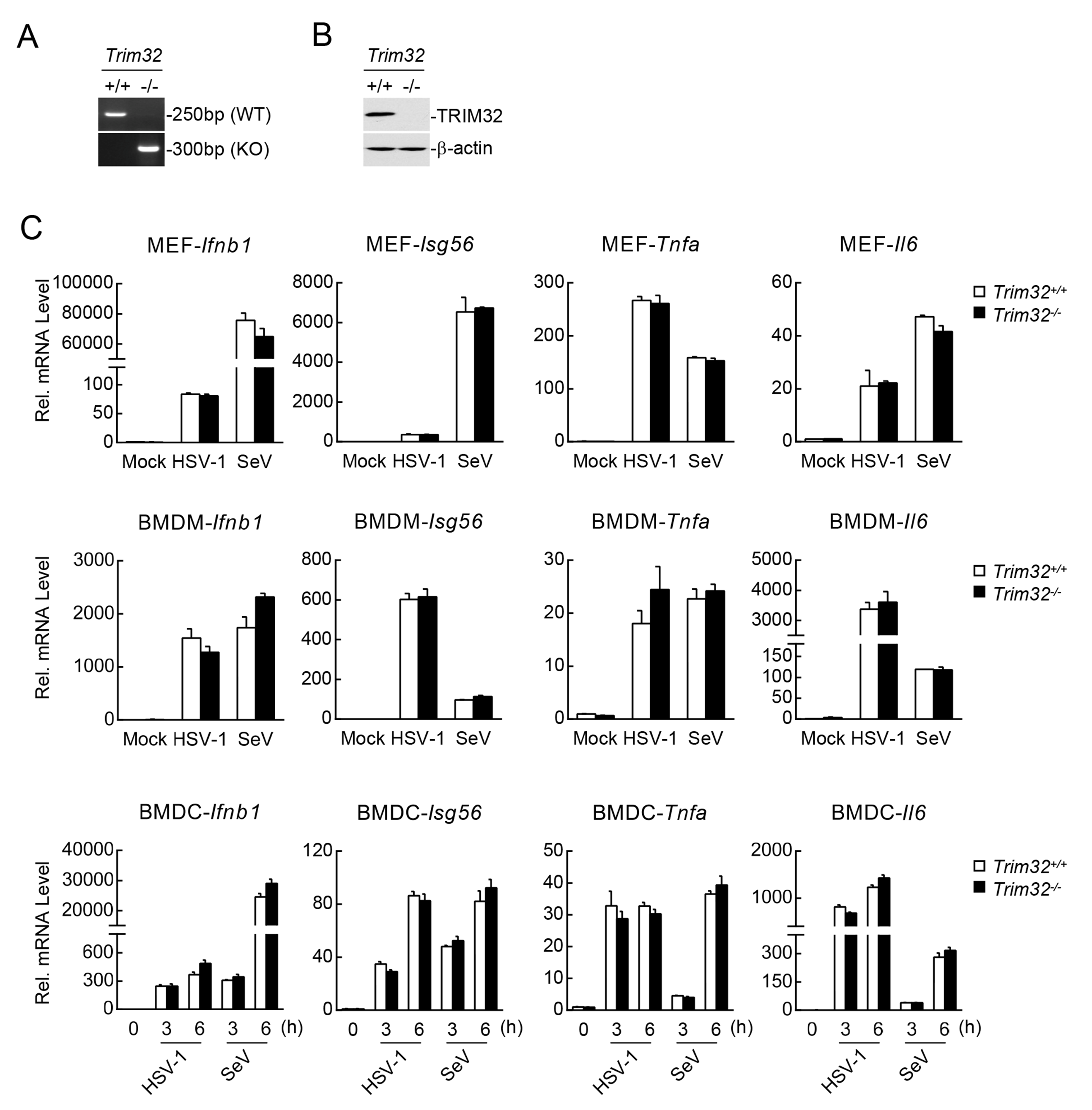

Supplement: S1 Fig — (A) PCR genotyping of Trim32 gene knockout mice. (B) Detection of TRIM32 in Trim32+/+ and Trim32-/- cells. Trim32+/+ and Trim32-/- cells were lysed followed by immunoblotting analysis with the indicated antibodies. (C) Effects of TRIM32-deficiency on SeV- and HSV-1-induced transcription of Ifnb1, Isg56, Tnfa and Il6 in MEFs, BMDMs and BMDCs. Wild-type and Trim32-/- cells were infected with SeV or HSV-1 before qPCR analysis. (TIF) [file ppat.1006600.s001.tif]

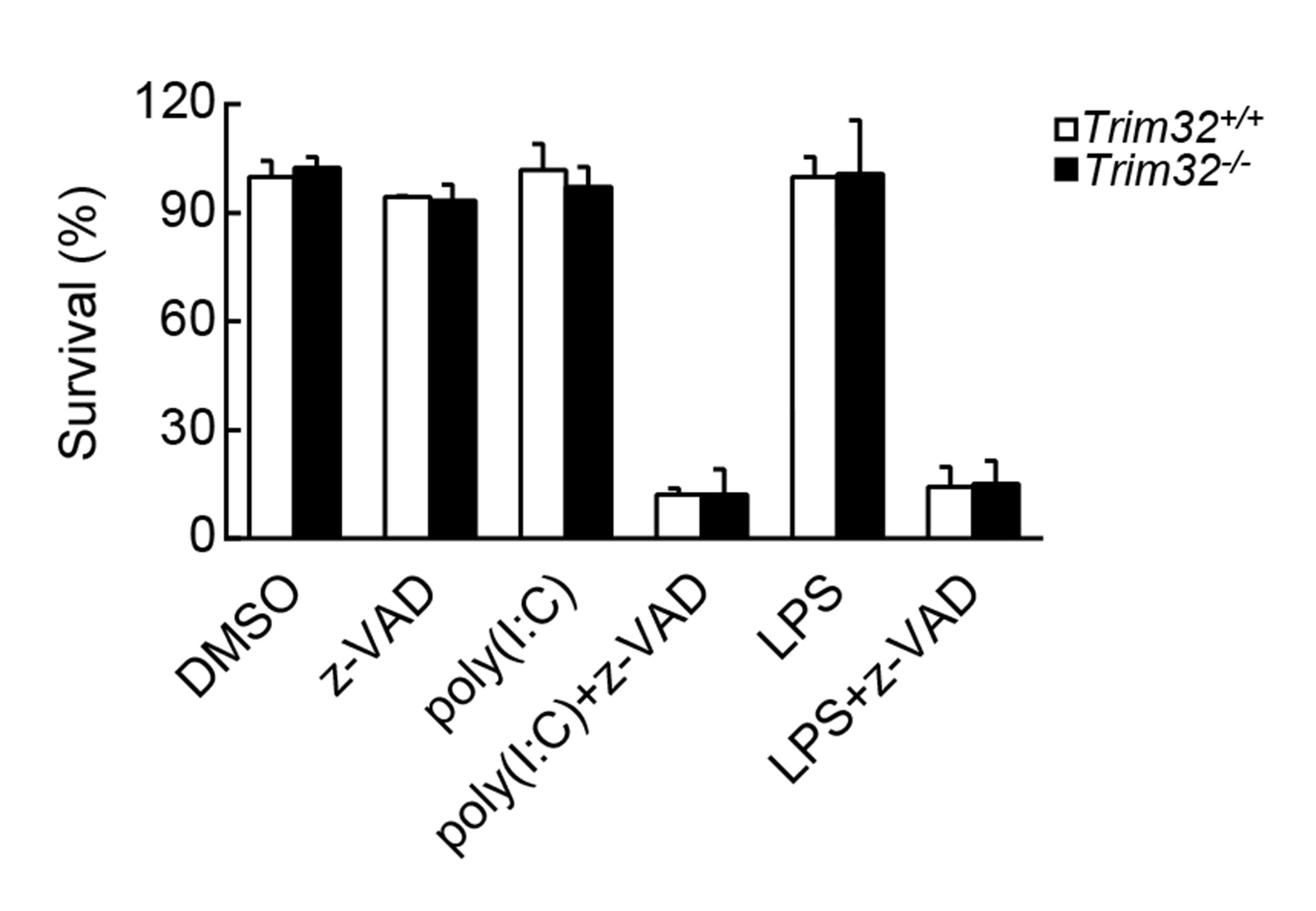

Supplement: S2 Fig — Trim32+/+ and Trim32-/- BMDMs were treated with DMSO, z-VAD (10 μM), poly(I:C) (50 μg/mL), poly(I:C) (50 μg/mL) plus z-VAD (10 μM), LPS (20 ng/mL) or LPS (20 ng/mL) plus z-VAD (10 μM) for 24 hours before cell viability assays. (TIF) [file ppat.1006600.s002.tif]

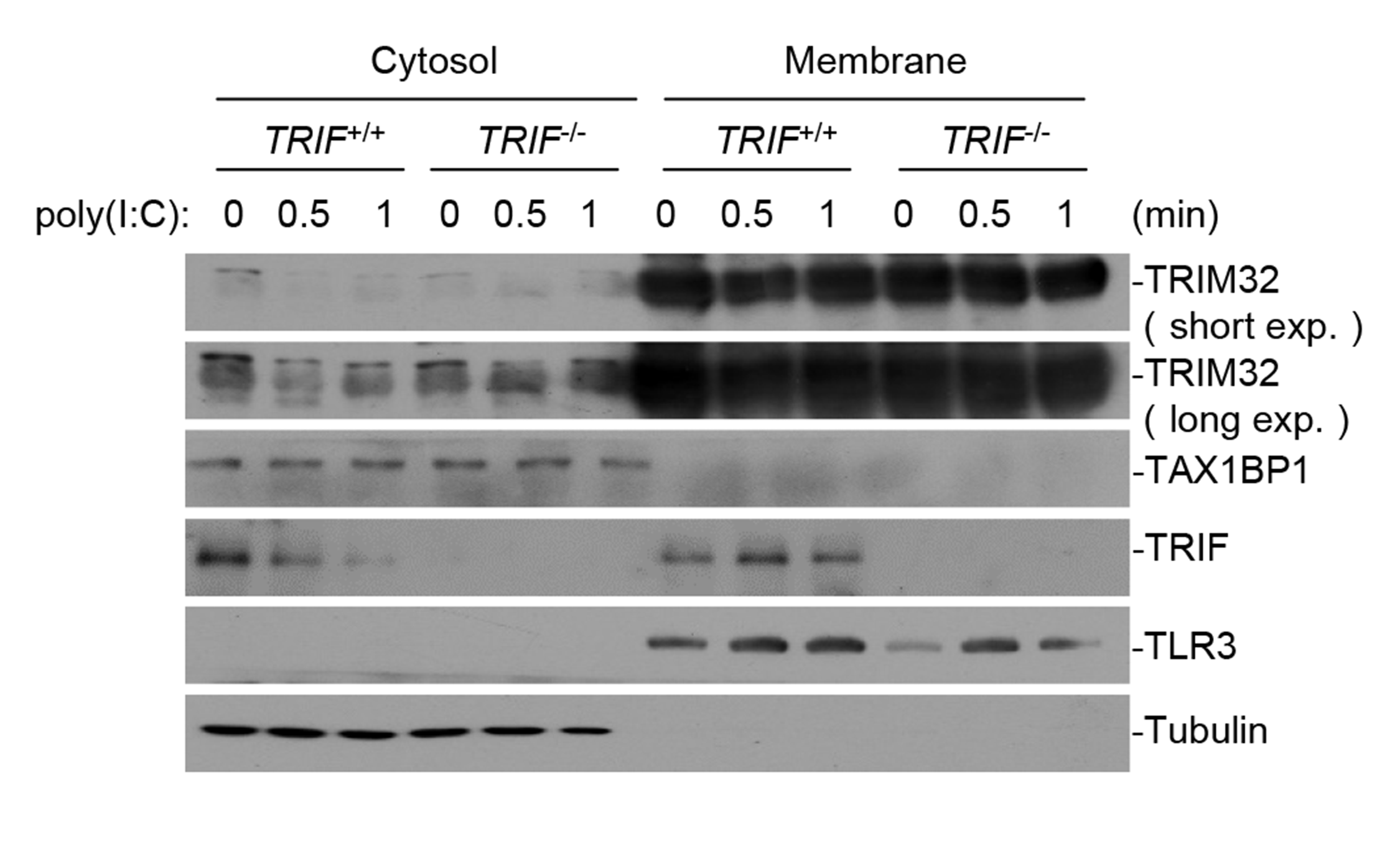

Supplement: S3 Fig — TRIF+/+ and TRIF-/- HEK293-TLR3 cells were treated with poly(I:C) (50 ng/mL) for the indicated times before cell fractionation and immunoblotting analysis with the indicated antibodies. (TIF) [file ppat.1006600.s003.tif]
